# A Novel, Low Computational Complexity, Parallel Swarm Algorithm for Application in Low-Energy Devices

**DOI:** 10.3390/s21248449

**Published:** 2021-12-17

**Authors:** Zofia Długosz, Michał Rajewski, Rafał Długosz, Tomasz Talaśka

**Affiliations:** Faculty of Telecommunication, Computer Science and Electrical Engineering, Bydgoszcz University of Science and Technology, 85-796 Bydgoszcz, Poland; michalrajon@gmail.com (M.R.); rafal.dlugosz@pbs.edu.pl (R.D.); tomasz.talaska@pbs.edu.pl (T.T.)

**Keywords:** solutions for swarm algorithms, deterministic approaches, low-energy devices, implementation in CMOS technology

## Abstract

In this work, we propose a novel metaheuristic algorithm that evolved from a conventional particle swarm optimization (PSO) algorithm for application in miniaturized devices and systems that require low energy consumption. The modifications allowed us to substantially reduce the computational complexity of the PSO algorithm, translating to reduced energy consumption in hardware implementation. This is a paramount feature in the devices used, for example, in wireless sensor networks (WSNs) or wireless body area sensors (WBANs), in which particular devices have limited access to a power source. Various swarm algorithms are widely used in solving problems that require searching for an optimal solution, with simultaneous occurrence of a different number of sub-optimal solutions. This makes the hardware implementation worthy of consideration. However, hardware implementation of the conventional PSO algorithm is challenging task. One of the issues is an efficient implementation of the randomization function. In this work, we propose novel methods to work around this problem. In the proposed approach, we replaced the block responsible for generating random values using deterministic methods, which differentiate the trajectories of particular particles in the swarm. Comprehensive investigations in the software model of the modified algorithm have shown that its performance is comparable with or even surpasses the conventional PSO algorithm in a multitude of scenarios. The proposed algorithm was tested with numerous fitness functions to verify its flexibility and adaptiveness to different problems. The paper also presents the hardware implementation of the selected blocks that modify the algorithm. In particular, we focused on reducing the hardware complexity, achieving high-speed operation, while reducing energy consumption.

## 1. Introduction

Algorithmic solutions with low computational complexity has gained importance and applicability. For example, various types of portable devices and processing units with limited energy sources benefit from bespoke implementations. These include sensors used in wireless sensor networks (WSNs), in which the collected data are sent to a base station or other central computing unit for further processing. In WSNs, one of the key issues is the possibility of operating such devices with low energy consumption, which translates into the operability without the need to replace the battery or the possibility of their operation based on energy scavenged from the environment. A vast amount of optimization work has been devoted to reducing the computational complexity of particular blocks of such devices (filters, analog-to-digital converters, etc.), in order to increase the ratio of computing power to consumed energy [1,2,3].

In conventional wireless sensors, most of the collected data are transmitted to a base station for a further analysis. In this case, the problem is that the radio-frequency (RF) communication block of the sensor may consume up to 90 % of total energy [4,5,6]. One of possible solutions to this problem is a reduction in the amount of transmitted data. It can be realized by placing selected data processing tasks directly at the sensor level and by triggering communication with the base station only when needed. We proposed one solution for such a problem in [7]. A similar approach may also be applied to artificial intelligence (AI) algorithms, which are frequently used in the process of analysis and inference on the basis of data provided by a group of sensors in the WSN [8,9]. In our previous work, we proposed solutions that enable the development of low energy consumption miniature artificial neural networks in specialized integrated circuits realized in CMOS technology [6,10,11].

The described possibilities fit into the topic known as edge computing, which has been intensively developed in recent years [12,13]. The goal is to process as much data as possible on site without having to send them to the base station. The use of such solutions can be found in agriculture [14]; healthcare [15]; as well as automotive industries, for example, in the developed of vehicle-to-infrastructure (V2I) communication technology [16].

The industry has many optimization problems that require fast and effective computational algorithms which operate regardless of the size (dimensionality) of the solution space. In the literature, one can find various algorithms that are used for optimization tasks. One such group is metaheuristic algorithms inspired by the observation of nature. This group contains, among others, genetic and swarm algorithms. The aim of the latter group is to describe the behavior of the herd members using a mathematical apparatus. Examples include Ant Colony Optimization (ACO) [17], Artificial Bee Colony (ABC) [18], Bat Algorithm (BA) [19], Bacterial Foraging Optimization (BFO) [20] and Particle Swarm Optimization (PSO) [21].

In our work, we implemented metaheuristic algorithms at the transistor level. Algorithms in this group offer various computational complexities, which have different meanings depending on the application. For example, software implementation requirements for the computation time are often negligible, and the mathematical complexity of the algorithm can be compensated for by selecting a platform with an appropriate computing power. However, hardware implementation, due to aforementioned reasons, relies substantially on a reduced computational complexity. The described limitations motivate our focus on hardware implementable algorithms. To make an efficient hardware implementation possible, a first step is the analysis of the algorithms on the mathematical level in order to identify places that can be optimized/simplified.

One of the swarm’s algorithms is the PSO algorithm proposed by Kennedy and Eberhart in 1995 [21], described in detail in Section 3. Compared with other metaheuristic algorithms mentioned earlier, it is characterized by the simple mathematical description, which enables its efficient (i.e., fast and energy-saving) hardware implementation. It is worth noting that this algorithm consists of random number generation and the application of two mathematical formulas trivial for hardware implementation. This is undoubtedly a great advantage that puts this algorithm at the forefront of all metaheuristic algorithms, if we take into account its speed and efficiency.

In the case of the hardware implementation of the PSO algorithm, in which particular particles are realized as separate circuits working in parallel, one of the challenging operations is also the determination of the so-called global best value on the basis of personal best values of particular particles in a given iteration of the optimization process. To overcome this problem, in one of our former works [10], we proposed a circuit that can be used for this purpose. An experimental circuit of this type for 64 particles has been implemented by us in a prototype chip realized in CMOS 130 nm technology. The circuit has been successfully verified by means of laboratory tests. It is a fully parallel and asynchronous (clock-less) solution, which needs only a dozen or so nanoseconds to determine the smallest (or the largest) value regardless of the number of compared signals. It is a universal circuit that can be used in many other AI algorithms, for example to determine the winning neuron in Kohonen self-organizing maps. In this prototype chip, we also designed other components necessary to realize the PSO algorithm in hardware, such as multipliers, and summing and subtracting blocks. One of the problems that remains to be solved is the random block, which is the topic of this paper.

The structure of this article is as follows. Section 2 briefly presents a state-of-the-art study in key aspects related to the proposed solutions. It includes areas of the application of the PSO algorithm, ways to optimize its structure in order to facilitate its hardware implementation, as well as the realization of components responsible for generating random values commonly used in conventional swarm algorithms. Since our aim is the elimination of the operation of calculating random values, in the state-of-the art section, we also discuss existing algorithms known as deterministic PSO (DPSO). We also provide examples of hardware realization of inverse square root operation, as this operation is used in our algorithm. The proposed solution has evolved from the conventional PSO algorithm. For this reason, Section 3 has been devoted to an explanation of the structure of this algorithm. Then, in Section 4, we provide details of the proposed modifications, as well as the proposed transistor level implementation of selected components of the algorithm. The results for the software level simulations as well as for the transistor level verification are presented in Section 5. These results are further discussed in Section 6. Conclusions are formulated in the last section.

## 2. State-of-the-Art Study

The PSO algorithm has been widely described in the literature and used in various areas. This study can be viewed from different points of view. On one hand, we have various applications of this algorithm, showing its universality. The selected areas are presented below. In a further part of this study, we focus on the modifications of this algorithm proposed in the literature, with a particular focus on hardware implementation as well as on methods of implementation of a block that generates random values.

### 2.1. Application Abilities of the PSO Algorithm

In [22], the PSO algorithm was used in flood protection systems. The discharge flow process for the flood control was performed by opening and closing the reservoir floodgates. A significant problem here was how to determine optimal rules for the system operating during the flood period.

The work reported in [23] presents the application of the PSO algorithm in the problem of detection of intrusions in IT systems. Since the complexity of the IT systems increase, the intrusion detection systems (IDSs) need to be constantly developed. One of the problems is noisy data that may affect the performance of such systems. To overcome this problem, in the work presented in [23], the PSO algorithm was used to reduce the number of false alarms and to increase the detection rate.

In another work reported in [24], the PSO and the ABC algorithms were applied into the problem of cancer classification. The authors of this work proposed a Fast Correlation-Based Feature (FCBF) selection method to filter out irrelevant and redundant features from the used biomedical data set repository. For the cancer classification, they used a Support Vector Machine (SVM) algorithm [25,26] optimized by the use of the PSO algorithm combined with the ABC one.

The paper [27] presented many other interesting applications of algorithms that were based on swarm intelligence. It is worth noting that the use of swarm algorithms in the industry, medicine, and everyday life is constantly increasing.

One of the potential areas of application is automation and industrial electronics. These algorithms can be implemented, for example, in the form of specialized microelectronic devices (ASICs). Such devices can be used as intelligent sensors, which, due to their high energy efficiency, can work both stationary or as mobile devices. One of the advantages of wireless sensor networks, in which particular sensors would be equipped in hardware-implemented swarm algorithms, is the possibility of parallel work by such swarms of agents (in particular sensors), additionally with the possibility of their wireless communication with each other. In this approach, a set of smart sensors distributed in a given area would itself become a swarm, with increased data processing capabilities. Such solutions would be different from conventional ones, in which sensors only collect data and transmit it to the base station for further processing. Low-energy-consumption hardware-implemented swarm algorithms enable the realization of more autonomous sensors that would communicate with the base station only occasionally. In this case, the base station could serve, for example, as an arbitrator determining the global best values for the overall swarm. It needs much less data to be exchanged between sensors and the base station—only single variables for each iteration of the algorithm. As a result, the overall WSN would consume much less energy.

The ability to implement the swarm algorithm in hardware is also useful in optimization problems that are computationally intensive or have nonlinear constraints. Descriptions of various applications of the PSO algorithm can be found, among others, in [28,29]. As described in these works, the PSO algorithm is used in the optimization of power system problems, control system design, or transportation network design. The algorithm also turns out to be very useful in the process of training artificial neural networks. The often slow and long-lasting learning process may be sped up by the PSO algorithm, used in this case to find the most favorable settings in the space of the network parameters. An example of such an application can be found in [30], where the PSO method is used to optimize weights and thus complements the traditional backpropagation algorithm.

### 2.2. Optimization Aspects of the PSO Algorithm

Various methods are proposed to optimize the PSO algorithm for specific practical applications. One example of such an optimization is the modification of the algorithm’s behavior as it approaches an optimal position [31]. Another example of the modification of the algorithm is to optimize the convergence speed of the algorithm [32].

The behavior of particular agents determines the performance of the overall PSO algorithm. One can modify such parameters as initial distribution of particles, factors impacting their velocities, as well as their interaction with other agents in the swarm. In [33], for example, the agents are mixed several times and divided into smaller groups (sub-swarms). This approach supports sharing the information between particles, enabling a faster descent to the minimum. Another work, Ref. [34], investigated the impact of the inertia mass and the imposition of the maximum velocity of particles on the efficiency of the overall algorithm. Similar studies were carried out in [35], where both aspects were compared—the effect of using inertia mass and imposing restrictions on particle movement. After examining several functions, it was found that constraining the maximum particle velocity works the best.

The literature also includes works examining the PSO algorithm from the theoretical point of view and by verifying its mechanisms. An example of such research is the work in [36], where the structure of the algorithm, the selection of parameters, and its discrete and parallel versions were tested.

There have also been attempts to simplify the PSO algorithm in terms of matching it to hardware. The authors of [37] noticed the performance problem of the classic software approach in the PSO algorithm, and for this reason, they undertook the development of its architecture so it is suitable for implementation in hardware. The aim was to speed up the computation for use in real-time applications. In addition to increasing the speed of computation, it is also more flexible, offers modularity, and is therefore more adaptable to other applications.

In [38], the authors continued to work on the hardware implementation of the PSO algorithm. Although compared with other population-based algorithms, PSO is considered an efficient algorithm, in some applications, its performance in multidimensional space needs further improvements and enhancements. The work in [38] extends the research of [37] and presents further improvements to the algorithm by introducing parallelism in it, which speeds it up and expands its applications in real-time applications.

### 2.3. Block Generating Random Values

In the examples discussed above [28,29,30], various modifications of the classic PSO algorithm have been applied. However, a component representing the random function is still in use in these works. Parallel implementation of the PSO algorithm in hardware requires that each particle in the swarm is equipped with its own circuit responsible for the generation of random numbers in particular iterations, *k*. In the literature, one can find various hardware realizations of such components. One of very popular methods is based on ring oscillators, which are based on the so-called, true random number generators (TRNG) [39,40,41]. Such generators are in turn based on rings of an odd number of the NOT logic gates as well as other supporting digital elements such as XOR gates, D-flip flops (DFF), etc. Such circuits usually feature a complex hardware structure. Additionally, TRNGs described in the literature usually allow us to generate only single bits of a digital signal. This means that to draw a multi-bit $r_{1}$ and $r_{2}$ signals for each component of the velocity vector *V* (refer to details provided in next Sections) requires a substantial serialization of the operations or the use of a number of such circuits working in parallel.

The random functions can also be realized in different ways. One of them is the circuit with a noise generator. The noise signal is amplified and then fed to the input of the comparator [42,43] or synchronous flip-flops [44]. At the outputs of comparators or flip-flops appears a random bit stream. The disadvantage of such solutions is the need to use clock signals, which makes it much more complicated if there is a need to use multiple generators at once. Additionally, in order to improve the quality of random signals, it is required to use auxiliary systems, such as an offset control system (using a DAC converter and a Voltage Ramp Generator). Another example of building a random number generator is the use of a chaos generator (such a solution is shown in [45]). The random number generator exploits a continuous-time chaotic circuit as the entropy source. A source-coupled multivibrator is used to transform the generated chaotic signal into jittered oscillations required in the oscillator sampling method.

Taking the complexity of the random function block into account, in this work, we focus on such modifications of the PSO algorithm that eliminate the necessity of using the random function. Since we eliminate the randomization, which is one of the characteristic operations for the PSO algorithm, we do not refer to our approach as a PSO algorithm, but only that we are based on this algorithm. We refer to the proposed swarm algorithms as deterministic ones. It is worth emphasizing that the goal of our work is to obtain a computationally simple solution that can be easily implemented in CMOS technology in devices that require low energy consumption. At the same time, however, we look for solutions that do not substantially differ in behavior from the original PSO algorithm.

### 2.4. Development of Deterministic PSO Algorithms

The methods of simplifying the implementation of the PSO algorithm also include deterministic solutions, in which randomness is replaced with predetermined trajectories of particular particles in the swarm. Selected works of this kind are discussed below.

In a series of papers [46,47,48,49,50] by Kenya Jin’no, Takuya Shindo, and others, a concept of DPSO algorithms was proposed and discussed in the context of a possible hardware implementation. It also shows the importance of such works. The authors of the aforementioned works noticed the limitations posed by the randomness of the traditional PSO. The lack of stochasticity is obtained in the following way. First, the values of $r_{1}$ and $r_{2}$ (see the details in the next section) are set to fixed values, thus in practice being eliminated. Then. the eigenvalues of the equations of velocity and particle position are computed. The use of these eigenvalues is used to provide spiral trajectories of particular particles in data space. If the conditions available, for example, in [49], are met, spiral movement obtains the desired results, thus reducing the need for random factors. As the authors mentioned, unfortunately, such a change has a negative effect on performance in relation to the original algorithm. The authors checked different values of the angle of rotation of the particles and the damping value of their motion. They chose these values in such a way that the particles do not follow the same trajectory but spread across space, thus effectively exploring it.

Another work of this type was presented in [51], where the authors presented a slightly different approach to the realization of the DPSO algorithm and tailored the solution to a specific problem. The PSO algorithm itself was used here to find the maximum power point in a system. The implementation of the algorithm in this solution was strongly based on the characteristics of the problem of systems operating with photovoltaic cells. Thanks to such adjustment to the problem, it is possible to optimize the algorithm in the most effective way, but at the same time, the universality of the solution is lost. Due to the knowledge of the system and the possibility to initially determine where the optimum is located, it is possible here to simplify the PSO algorithm and to remove the random element, thanks to which the algorithm becomes deterministic.

The proposed solution introduces a deterministic approach as well. The modification of the conventional algorithm as well as the proposed corresponding hardware solutions significantly minimized the complexity of additional blocks that replace the random ones. Compared with the first of the state-of-the-art solutions presented above, in our solution, there is no need for matrix calculations (eigenvalues) and for computing the values of trigonometric functions. The proposed structure of the algorithm allowed us to limit the complexity to simple mathematical operations only.

### 2.5. Solutions for Inverse Square Root Operation

In the proposed approach, an inverse square operation is used. For this reason, the implementability of this operation in hardware is the main subject of this section. Square root operations can be performed either analogously or digitally. In the first case, this operation is performed using the current [52,53], voltage [54], or mixed mode [55]. One of the popular solutions of square root circuits operating in voltage mode is their implementation with the use of multipliers and operational amplifiers. An operational amplifier working in the inverting configuration [54] is placed in the negative feedback loop. In addition, the multipliers and operational amplifiers enable the division operation, which makes it relatively easy to perform the $1/\sqrt{x}$ operation.

Since in this paper we deal with digital implementation, it is more appropriate to take a closer look at existing solutions in digital technique. An example is a solution presented in [56], which implements the square root function and inverse square root in Field Programmable Gate Arrays (FPGAs). In that paper, a modified Quake’s algorithm was used. The authors of [56] proposed a solution involving seven magic (base) numbers being stored in a look up table (LUT). The usage of these numbers allow for faster computation of square root ($y=\sqrt{x}$) and inverse square root ($y=1/\sqrt{x}$) functions. This solution offers an accuracy up to 12 bits in all cases of the IEEE754 single-precision floating point numbers. The output values of both functions are ready after 12 clock cycles, at the sampling frequency of about 194 MHz.

Another approach to hardware implementation of the square-root and the inverse square root functions was proposed in [57]. The circuits based on seed generators were designed in CMOS 45 nm technology. The authors proposed three low-cost realizations. In one of them, the square root was computed with the use of the iterative Newton–Raphson (NR) method. It is able to achieve the precision of 20 bits. The remaining two solutions allow for computing the inverse square root with precisions of 4 and 5 bits. The circuits have been designed for the application in embedded systems with limited area resources, where high precision is not required. Example applications include sensor networks used in internet-of-things (IoT) systems and in parallel computing in various AI algorithms.

Another circuit realized in CMOS technology is proposed in [58]. It deals with the computation of several functions that include reciprocals, square roots, inverse square roots, logarithm, and exponential ones. The authors of this work compared their proposed method with other state-of-the art solutions, which include the iterative NR method mentioned above as well as those proposed for example by Wong and Goto [59] and by Ito, Takagi, and Yakima [60]. All of these methods were developed in terms of the possibility of obtaining very high computation precision (double precision). They are based, inter alia, on multipliers in which the numbers to be multiplied have a resolution of up to several dozen bits. For this reason, these circuits are very complex in terms of hardware. A single multiplier for the numbers with such high resolutions requires the use of several tens of thousands of transistors. For comparison, in the modification of the PSO algorithm proposed by us, the inverse square root is calculated only for a dozen or so natural numbers, which are the following powers of the number 2. For this reason, we used a much simpler approach from the hardware point of view, which requires the use of only a few hundred transistors.

## 3. An Overview of a Conventional PSO Algorithm

The PSO algorithm is a stochastic computing algorithm. It works by mimicking the behavior of a moving flock of birds. All herd individuals (so-called particles), in order to be able to find food, move at a speed calculated on the basis of their own best experiences as well as the best experiences of the rest of the herd. In other words, the PSO algorithm searches a space with particles in which the velocity and position are modified using simple mathematical formulas. Doing so moves the particles towards the best solutions. The particle swarm algorithm has many similarities to evolutionary computing techniques such as genetic algorithms. However, in contrast, PSO does not use the often complex computation of evolutionary operators such as, f selection, crossing, and mutation.

The algorithm proposed in this paper is largely based on the PSO algorithm. For this reason, in this section, we provide the basics of the latter one in order to illustrate the differences.

The PSO algorithm has been widely used for many years. Through the modeling of the behavior of flocks of animals, e.g., ants or bees, it offers a good performance in problem solving tasks that require searching for a global optimum in situations where many local extrema exist. Particular agents in the network cooperate with each other and share the acquired knowledge with the swarm. The way the agents change their locations is to some extent similar to how animals move in nature. Every particle in the swarm is characterized by several basic variables:

- position—position of a particle in search space;
- velocity—speed of a given particle;
- $p_{\mathrm{BEST}}$—personal best value found so far by a given agent and its position; and
- $g_{\mathrm{BEST}}$—global best value found so far by any agent in the swarm and its position.

One of the key indicators for assessing the behavior of the swarm is the so-called fitness function (FF). This function defines a problem that needs to be solved by determining where a global extreme is located in data space.

Before starting an iterative part of the PSO algorithm, it is necessary to initialize the positions of particular particles in the swarm, *X*, and their starting velocities, *V*. In the original PSO algorithm, the values of these parameters are chosen in a random fashion. For comparison, in the proposed deterministic solution, the stage of pre-randomizing the positions of the particles may be omitted, which is an advantage, as it simplifies the hardware implementation.

Both drawing operations of the *X* and the *V* vectors are relatively complex from the point of view of the hardware implementation. For this reason, we investigated alternative ways, verifying their impact on the effectiveness of the optimization process of the swarm. One of the investigated options was setting the starting values of the velocities to zero, while the positions were selected so that the swarm covers the overall data space. This aspect is a separate problem and thus is not investigated in this work in more detail.

The process of the optimization of the swarm is performed iteratively. In this paper, each iteration, *k*, is referred to as an epoch that is further divided into steps, described below for a conventional version of the PSO algorithm.

### 3.1. Computation of the Fitness Function

In every iteration *k*, the first step is to calculate the value of the FF for each particle, based on the positions of these particles in an *n*-dimensional search space. The FF determines the problem that the PSO algorithm has to solve. This function can be both provided as a mathematical expression, as for example in [61,62,63,64] or as a real world situation, where its value may be determined by measurements of different physical quantities [65,66]. However, regardless of the approach, the values that are returned by the FF show how well particular particles match the optimal solution, i.e., how close they are to an optimal value.

### 3.2. Updating Personal Best Values

In the following step of the algorithm, each particle in the swarm updates its own personal best value, $p_{\mathrm{best}}$. If the value of the FF, computed in a given iteration *k*, is better than its previous value, the $p_{\mathrm{best}}$ is replaced with just the computed output of the FF. The positions for which the best values were computed are also stored in the memory blocks of particular particles. The term ‘better’ means either a smaller or larger value, depending on the problem.

### 3.3. Updating Global Best Value

After updating the personal best values, the next step is to determine which of them is the best one (an optimum), which in practice means it is the closest to a given extreme of the FF. This value is then stored in the memory of the swarm, as the global best value, $g_{\mathrm{best}}$. Together with the value of $g_{\mathrm{best}}$, the position for which it has been found is also stored, as in the case of $p_{\mathrm{best}}$ variables of particular particles.

### 3.4. Updating Particle Velocities

On the basis of the updated values of $p_{\mathrm{best}}$ and $g_{\mathrm{best}}$ factors, in the following step, the algorithm updates the velocities and the positions of all particles in the swarm. First, it updates the velocity vectors of particular particles, which are composed of three components: (i) the inertial, (ii) the cognitive, and (iii) the social ones [21,36], as discussed below.

#### 3.4.1. Inertial Component

To compute the inertial component, the velocity vector, *V*, taken from the previous iteration, k−1, is multiplied by a weights vector *W*. However, the elements of *W* can be equal, which in practice means that *V* is multiplied by a scalar factor. In the conventional PSO algorithm, the elements of *W* are gradually decreased during the optimization process. The velocity component is computed as follows [67]:

(1) $$V_{\mathrm{ic},k}=W_{k}\cdot V_{k}$$

#### 3.4.2. Cognitive Component

The cognitive velocity components are computed based on the positions, for which particular particles found their personal best values, $p_{\mathrm{best}}$. The component is computed as follows [67]:

(2) $$V_{\mathrm{cc},k}=c_{1}\cdot r_{1,k}\cdot \left(p_{\mathrm{best}}.X_{k}-X_{k}\right)$$

#### 3.4.3. Social Component

The social component is dependent on the position, for which the global best value, $g_{\mathrm{best}}$, of the overall swarm was found. The component is computed as follows [67]:

(3) $$V_{\mathrm{sc},k}=c_{2}\cdot r_{2,k}\cdot \left(g_{\mathrm{best}}.X_{k}-X_{k}\right)$$

The $p_{\mathrm{best}}.X$ and $g_{\mathrm{best}}.X$ variables in Equations (2) and (3) store the positions for which personal and global best values were found, respectively. The resultant velocity is the sum of the three components described above [67]:

(4) $$V_{k}=V_{\mathrm{ic},k}+V_{\mathrm{cc},k}+V_{\mathrm{sc},k}$$

In the conventional PSO algorithm, the cognitive and the social velocity components are computed with the use of two random vectors, $r_{1}$ and $r_{2}$.

### 3.5. Updating Particle Positions

Based on the velocities computed with the formulas presented above, in the following step, the particle positions are updated, as follows [67]:

(5) $$X_{k+1}=X_{k}+V_{k}$$

where:

- $X_{k}$—position of a given particle in a given iteration;
- $V_{k}$—velocity of a given particle in a given iteration;
- $W_{k}$, $c_{1}$, $c_{2}$, $r_{1,k}$, $r_{2,k}$—parameters that play the role of the weights.

### 3.6. Final Step—Terminating the Optimization Process

The optimization process of the swarm may be considered complete and thus terminated based on several criteria. One of them is an arbitrary assumption of a maximum number of the iterations. In an alternative approach, the optimization process may be terminated when the values of personal best and global best variable stop changing or only change very little.

A general objective of the optimization process is to determine the position, for which the FF achieves its global extreme. It is often desired that all particles in the swarm are located in close proximity to this point. However, in some scenarios, it may happen that only selected particles reach this position or oscillate around it. In real problems, it may happen that the FF has many local extremes in the input data space. In this situation, particular particles can become representatives of them.

## 4. Materials and Methods

### 4.1. Materials/Tools

The proposed swarm algorithm has been initially implemented in the Matlab/Octave environment and compared with the original PSO algorithm. This environment allows for fast prototyping of new solutions, facilitates introducing corrections as well as generating simulation results with a convenient way of their visualization. The program has been written in a way that enables its quick implementation in lower-level languages such as C/C++. Using this model, all tests were carried out to verify the algorithm in terms of correctness of operation for various classes of the problem. These classes have been expressed in terms of various fitness functions typically used to verify swarm algorithms. During the work on the idea, attempts were made to use functions in which there are many local optima or in which the optimum is strongly blurred. Details are provided in the Results section below.

Part of the work was also carried out in the Hspice environment, which was used to simulate electronic circuits. This was due to the main goal of the research, which is the hardware implementation of the proposed deterministic swarm algorithm. At this stage, the correct operation of individual electronic blocks used in the hardware version was checked. The goal of this was also to check the proposed concept of selected units in terms of their operation speed and energy consumption.

### 4.2. Methods—Proposed Algorithms

The complexity of a hardware implementation mostly depends on the complexity of the mathematical operations necessary at each stage of the calculations. In the case of implementation at the transistor level, the increase in computing power is possible thanks to the implementation of parallel signal processing. In practice, however, it is not always the optimal solution from the hardware complexity point of view. We propose a solution in which a significant part of the operation is performed in parallel. In the proposed approach, each particle in the swarm is a separate system that performs in a parallel manner to those operations that concern itself. In the PSO algorithm, the only step in which the particles must communicate with each other is to determine the global best value. This operation requires comparing the instantaneous $p_{b}est$ values of the individual particles in order to find the smallest value. This value becomes the new global best value of the entire swarm.

In the proposed approach, in order to maintain low hardware complexity, despite the parallelization of calculations, it was necessary to simplify the computational complexity of individual stages of the algorithm itself. In the conventional PSO algorithm, the values of $r_{1}$ and $r_{2}$ coefficients were randomly generated in each iteration, *k*, for each particle in the swarm. As a result, the complexity of the block responsible for generating these values has a significant impact on the complexity of the entire network. It is important to remember that each of the particles generates its own random values of these coefficients. As a result, theoretically, each of the particles should be equipped with its own hardware block for determining their value. Taking this into account, in our work, we focused on such modifications of the PSO algorithm that eliminate the need to use a random block. In our work, we focused on replacing randomization with more deterministic methods. We proposed a mechanism that allows us to differentiate the trajectories of individual particles, using a much simpler hardware solution. As a result, individual particles occupy a smaller area in the integrated circuit and consume less energy.

### 4.3. Proposed Modifications of $r_{1,2}$ Coefficients

The modification of the $r_{1}$ and $r_{2}$ parameters impacts the velocity components represented by Equations (2) and (3). Below, we provide details of the proposed modifications of the $r_{1,2}$ coefficients.

In the proposed method, the random coefficients $r_{1}$ and $r_{2}$ were substituted by deterministic values that are functions of the square root of the epoch number, *k*, as expressed below:

(6) $$r_{1,2}\left(k\right)=\frac{r_{\mathrm{base}}}{\sqrt{k}}$$

In this approach, with each following iteration, *k*, the velocity with which the particle position is updated is gradually decreased. This procedure allows us to avoid oscillations closer to the end of the overall optimization process. As a result, in the following iterations *k*, the search area for particular particles is gradually reduced. This allows for finding the optimum more precisely. The $r_{\mathrm{base}}$ factor is used to control the course of the algorithm. It is usually a small fixed point number. This factor may additionally differ for particular particles.

In Equation (6), the difficulty from the hardware point of view is the need to calculate the root for each iteration. Additionally, the expression $\sqrt{k}$ is in the denominator, which requires the division operation to be performed.

Theoretically, this algorithm can be further simplified by eliminating the square root operation, as follows:

(7) $$r_{1,2}\left(k\right)=\frac{r_{\mathrm{base}}}{k}$$

As a result, we obtain two variations of this algorithm. Both of them were verified and compared by simulations carried out in the software model of the algorithms, implemented in Matlab. The results of these simulations are presented in the following section.

In the next step of our investigations, we considered a possibility of a further modification of both variants of the algorithm described by Equations (6) and (7). The goal was to simplify their hardware implementation. In the adopted solutions, the *k* variable in the denominator was replaced with a new variable $D_{k}$. The proposed modification relies on an iterative modification of this variable, as presented below. Depending on the approach, the value of this variable is initially (for k=1) set to a small number, $D_{1}=\left\{1,2,4,\;\mathrm{or}\;8\right\}$. Then, for each new iteration, the $D_{k}$ variable is compared with the *k* variable, and this variable is updated as follows:

(8) $$\mathrm{if}\;\left(k>D_{k}\right)\left\{D_{k+1}=D_{k}\cdot 2;\right\}$$

As a result, the value of the $D_{k}$ factor equals one of the powers of 2, i.e., $D_{k}\in 2,4,8,16,32,\ldots$. The increase in the value of this variable can be accomplished by shifting the bits to the left by a given number of positions, as follows:

(9) $$\mathrm{if}\;\left(k>D_{k}\right)\left\{D_{k+1}=D_{k}\;«\;1;\right\}$$

Introducing the $D_{k}$ variable allows for the modification of Equations (6) and (7), respectively, as follows:

(10) $$r_{1,2}\left(k\right)=\frac{r_{\mathrm{base}}}{\sqrt{D(k)}}$$

(11) $$r_{1,2}\left(k\right)=\frac{r_{\mathrm{base}}}{D(k)}$$

In the case of the variant described by Equation (7), the problem of computing the square root does not occur. In this case, the implementation of the system requires only the operation of shifting bits by a certain number of positions, directly resulting from the value of the *D* variable.

There is still the problem of implementing the $1/\sqrt{\left(\right)}$ operation in the algorithm version described by the dependency (10). In this case, we introduced an additional variable to the numerator while eliminating the $\sqrt{\left(\right)}$ operation in the denominator. This made it possible to obtain approximate values for $\sqrt{D}$. As a result, Equation (10) takes the form:

(12) $$r_{1,2}\left(k\right)=r_{\mathrm{base}}\cdot \frac{C_{k}}{D_{k}}$$

In one of the proposed implementation options of Equation (12), the *C* vector composed of elements $C_{k}$ which correspond to particular values of $D_{k}$ variable, is stored in a Look-Up-Table (LUT). The application of the LUT is not a problem here for several reasons. First, the size of the *C* vector is small, as the number of distinct values of $D_{k}$ is also small. It approximately equals $\log_{2}k_{\max}$, where $k_{\max}$ is a total number of iterations of the optimization process of the swarm. For several thousand iterations, the size of the LUT does not exceed 10–12. The second reason is that the values of $D_{k}$ and $C_{k}$ are equal for all particles in the swarm. For this reason, a single LUT block may be required for the overall swarm. In this case, however, in the memory of each particle, it is necessary to store a specific value of the $r_{\mathrm{base}}$ factor, used in Equations (10) and (11). This factor is used to differentiate the values of resultant *r* variables for particular particles. Another option is to equip each $i^{\mathrm{th}}$ particle with its own LUT, with the values of $r_{\mathrm{base},i}\cdot C_{k}$ expression.

In this approach, to determine the inverse square root of $D_{k}$ variable and finally to perform the computation of the cognitive and the social velocity components, the values from the LUT after their multiplication by the $r_{\mathrm{base}}$ factors and by personal or global best values were then shifted by a given number of positions to the right (division by the $D_{k}$ variable). The bit shifting operation requires a commutation field composed of switches realized either by transmission gates or by a set of logic AND gates. For 12 distinct values of $C_{k}$ and $D_{k}$, the number of transistors in the commutation field does not exceed 310. This number is due to the fact that the 12 values stored in the LUT, after the mentioned multiplication, can be shifted by 1 to 12 positions. This requires 144 switches. Additionally, 11 switches are required in this case to connect outputs that due to bit shifting float to the ground. A single switch is realized as a transmission gate composed of two (NMOS and PMOS) transistors connected in parallel. In this approach, a fixed-point multiplication circuit is required, but each particle is already equipped with such a component, as it is also used in other operations.

To determine the values of the *C* vector, stored in the LUT, we start with following theoretical values of expression $1/\sqrt{D_{k}}$:

(13) $$G_{0}=\frac{1}{\sqrt{D}}\approx \left\{1.000,0.707,0.500,0.353,0.250,0.177,0.125,0.088388,0.0625,0.044194,\ldots \right\}$$

To approximate these values, one can use the following values of the variables $C_{k}$ and $D_{k}$:

(14) $$\begin{array}{ccc} C_{1,i} & = & \left\{8,\;\;11,\;\;16,\;\;23,\;\;32,\;\;45,\;\;64,\;\;91,\;\;128,\;\;181,\ldots \right\}\\ D_{1,i} & = & \left\{8,\;\;16,\;\;32,\;\;64,\;\;128,\;\;256,\;\;512,\;\;1024,\;\;2048,\;\;4096,\ldots \right\} \end{array}$$

The approximated values of the expression $1/\sqrt{D}$ are as follows:

(15) $$G_{1,k}=\frac{C_{1,k}}{D_{1,k}}\approx \left\{1.000,\;\;0.687,\;\;0.500,\;\;0.359,\;\;0.250,\;\;0.176,\;\;0.125,\;\;0.08987,\;\;0.0625,\;\;0.04419,\cdots \right\}$$

For larger values of $D_{1,k}$ and $C_{1,k}$ variables, the approximation error of $1/\sqrt{D_{k}}$ is below 1 %.

In the next step of our investigations, we checd the possibility of using more regular values of the $C_{k}$ variable:

(16) $$\begin{array}{cccccccccccccc} C_{2,k} & = & \{ & 2, & 3, & 4, & 6, & 8, & 12, & 16, & 24, & 32, & 48, & \cdots \}\\ D_{2,k} & = & \{ & 2, & 4, & 8, & 16, & 32, & 64, & 128, & 256, & 512, & 1024, & \cdots \} \end{array}$$

In this approach, for even values of $C_{k}$ and $D_{k}$ variables, the approximation error of expression $1/\sqrt{D}$ does not exceed 6 %. For odd values, however, the error equals 0 %. Both approaches have been successfully validated by means of the software model of the algorithm. In the remainder of this section, we present how to implement the $r_{1,2}$ variables in the second approach. In this case, there is no need to store the $C_{k}$ variables in the LUT. Both sets of variables listed in (16) can be expressed as follows:

(17) $$\begin{array}{cccccccccccccc} C_{2,k} & = & \{ & 2\cdot 1, & 3\cdot 1, & 2\cdot 2, & 3\cdot 2, & 2\cdot 4, & 3\cdot 4, & 2\cdot 8, & 3\cdot 8, & 2\cdot 16, & 3\cdot 16, & \cdots \}\\ D_{2,k} & = & \{ & 2, & 4, & 8, & 16, & 32, & 64, & 128, & 256, & 512, & 1024, & \cdots \} \end{array}$$

To explain the hardware implementation of such coefficients, one can express them as follows:

(18) $$\begin{array}{ccc} C_{2,k} & = & \left\{\mathrm{Bx}10\;«\;0,\;\;\mathrm{Bx}11\;«\;0,\;\;\mathrm{Bx}10\;«\;1,\;\;\mathrm{Bx}11\;«\;1,\;\;\mathrm{Bx}10\;«\;2,\;\;\mathrm{Bx}11\;«\;2,\;\;\mathrm{Bx}10\;«\;3,\;\;\cdots \right\}\\ D_{2,k} & = & \left\{\mathrm{Bx}10\;«\;0,\;\;\mathrm{Bx}10\;«\;1,\;\;\mathrm{Bx}10\;«\;2,\;\;\mathrm{Bx}10\;«\;3,\;\;\mathrm{Bx}10\;«\;4,\;\;\mathrm{Bx}10\;«\;5,\;\;\mathrm{Bx}10\;«\;6,\;\;\cdots \right\} \end{array}$$

**Figure 1:**
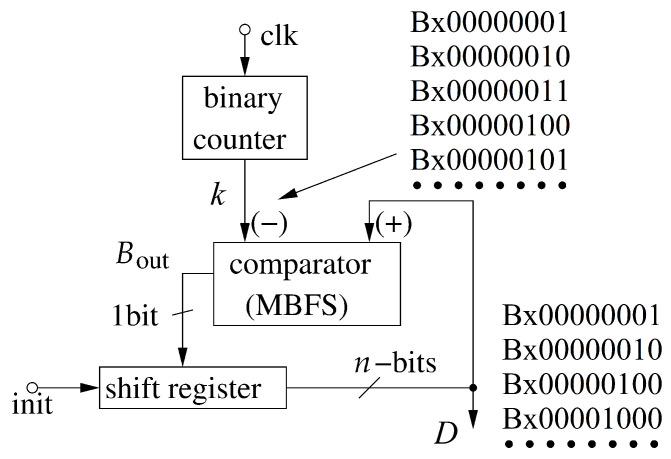
Proposed circuit used to switch the value of the denominator in order to control the course of the proposed deterministic algorithm.

In this case, the coefficients 2 and,3 which appear in Equation (17), are expressed as binary numbers Bx10 and Bx11, respectively. When we look at Equation (18), we can observe a regularity of changes in both variable data sets. For the $C_{k}$ variables, the multiplication by factors 2 or 3 is performed alternatively. For the multiplication by 2, the input value is only shifted by one position to the left. The multiplication by the value 3 is realized by a single summing operation of the input value with the same value after shifting it by one position to the left. This operation requires a single multi-bit full adder (MBFA) and a set of AND logic gates. One of the inputs of the MBFA receives a given input value after passing it by a block of two-input AND gates. Simultaneously, the same value, after shifting it by one position to the left (×2), is provided to the second input of the MBFA. The resultant value is further shifted by a given number of positions to the left, as in expression (18). As a result, expression (18) may be rewritten as follows:

(19) $$\begin{array}{ccc} C_{2,k} & = & F_{23}\;«\;b_{C,k}\\ D_{2,k} & = & D_{1}\;«\;b_{D,k} \end{array}$$

where $F_{23}=\mathrm{Bx}10$ or Bx11, while $b_{C,k}$ and $b_{D,k}$ are the numbers of positions by which the factors $F_{23}$ and $D_{1}$ are shifted in following iterations *k*. Taking into account expression (18), one can observe that $b_{C,k}$ increases 2× slower than $b_{D,k}$. To shift the outcome of the multiplication by the factor $F_{23}$ one can use a similar circuit (shown in Figure 1), as the one used to control the value of $D_{k}$ variable.

The way to implement the operation $D_{k+1}=D_{k}\;«\;b_{k}$ in hardware is shown in Figure 1. The variable $b_{k}$ is the number of positions by which the variable $D_{k}$ has to be shifted. The core block here is a digital comparator, built on the basis of a multi-bit full subtractor (MBFS) and a shift register. The shift register can be implemented either with a chain of D-flip flops (DFF) or as a chain of alternately connected NOT gates and switches, controlled by a two-phase clock. The shift register is directly controlled by the borrow-out bit, $B_{\mathrm{out}}$, returned by a 1-bit full subtractor (1BFS) that represents the most significant bit (MSB) of the MBFS. The resultant multi-bit variable $D_{k}$ controls the commutation field described above.

Initially the inputs (init terminal) of the shift register are set to a value dependent on the applied approach. For the values expressed by (14), the initial value is set to 8, while for the (16) approach, it is set to 2. The initial value of *k* is set accordingly, depending on the applied approach. This variable is provided to the negative input of the MBFS. During the optimization process of the swarm, the *k* variable is incremented in each iteration of the algorithm. When it becomes larger than the current state of the shift register representing the $D_{k}$ variable, the $B_{\mathrm{out}}$ bit becomes logical ‘1’. This signal triggers the operation of shifting all bits in the register by one position to the left (multiplication by 2).

From the practical point of view, it is necessary to demonstrate the implementation of the overall algorithm in the light of the proposed modifications. When we look at Equations (2) and (3), we can notice a series of multiplication operations. Since $c_{1,2}$ and $r_{\mathrm{base}}$ are constants for a given *i*-th particle, they can be combined into a single variable. Let us denote it as $\rho_{1,2,i}=c_{1,2}\cdot r_{\mathrm{base},i}$. In the first approach, in which the $C_{1}$ and $D_{1}$ variable sets (expressed by (14)) are applied, Equations (2) and (3) can be rewritten as follows:

(20) $$V_{\mathrm{cc},k}=\left(\left(p_{\mathrm{best}}.X_{k}-X_{k}\right)\cdot \rho_{1}\cdot C_{1,k}\right)\;»\;b_{D,k}$$

(21) $$V_{\mathrm{sc},k}=\left(\left(g_{\mathrm{best}}.X_{k}-X_{k}\right)\cdot \rho_{2}\cdot C_{1,k}\right)\;»\;b_{D,k}$$

On the other hand, if we use the $C_{2}$ and $D_{2}$ variable sets, Equations (2) and (3) can be rewritten as follows:

(22) $$V_{\mathrm{cc},k}=\left(\left(\left(p_{\mathrm{best}}.X_{k}-X_{k}\right)\cdot \rho_{1}\cdot \left(2\;\mathrm{or}\;3\right)\right)\;«\;b_{C,k}\right)\;»\;b_{D,k}$$

(23) $$V_{\mathrm{sc},k}=\left(\left(\left(g_{\mathrm{best}}.X_{k}-X_{k}\right)\cdot \rho_{2}\cdot \left(2\;\mathrm{or}\;3\right)\right)\;«\;b_{C,k}\right)\;»\;b_{D,k}$$

Equations (22) and (23) require only a single multiplication by a constant factor ρ, a single summing operation (representing the multiplication by the factor 2 or 3) and two bit-shift operations. The last two operations represent an additional multiplication and the division operations, respectively. Theoretically, both these bit-shift operations can be combined into a single one. This would require computing the $b_{D,k}-b_{C,k}$ term. Another option is to use two separate computation fields connected in a chain, with the first of them being controlled by the $b_{C,k}$ signal and the second one by the $b_{D,k}$ signal.

Based on Equations (22) and (23), one can estimate the hardware complexity of the proposed algorithm for the second approach, as follows. A single multiplier, e.g., 16×16, implemented as an asynchronous binary tree composed of 15 MBFAs of different lengths, requires approximately 12,350 transistors. The multiplier by the value 2 or 3 is realized as a single MBFA built of approximately 1150 transistors. This value is for a 32-bit input signal (output of the multiplier), including the AND gates described above. Then two commutation fields are required, each consisting of (32×12+11)×2=790 transistors. The computation of the $b_{C,k}$ and $b_{D,k}$ factors requires the circuits shown in Figure 1, each consisting of 700 transistors, as described above. The block responsible for calculating either the social or the cognitive component is therefore made up of around 16,500 transistors.

## 5. Results

### 5.1. Results at the Software/Model Level

**Figure 2:**
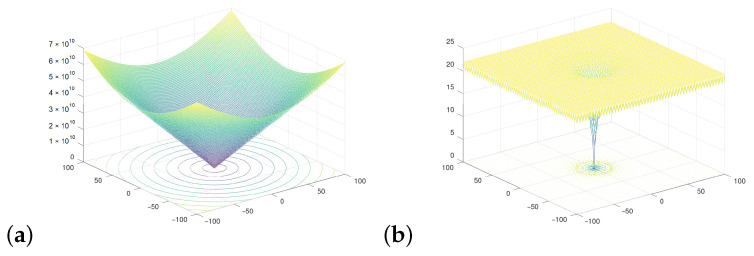

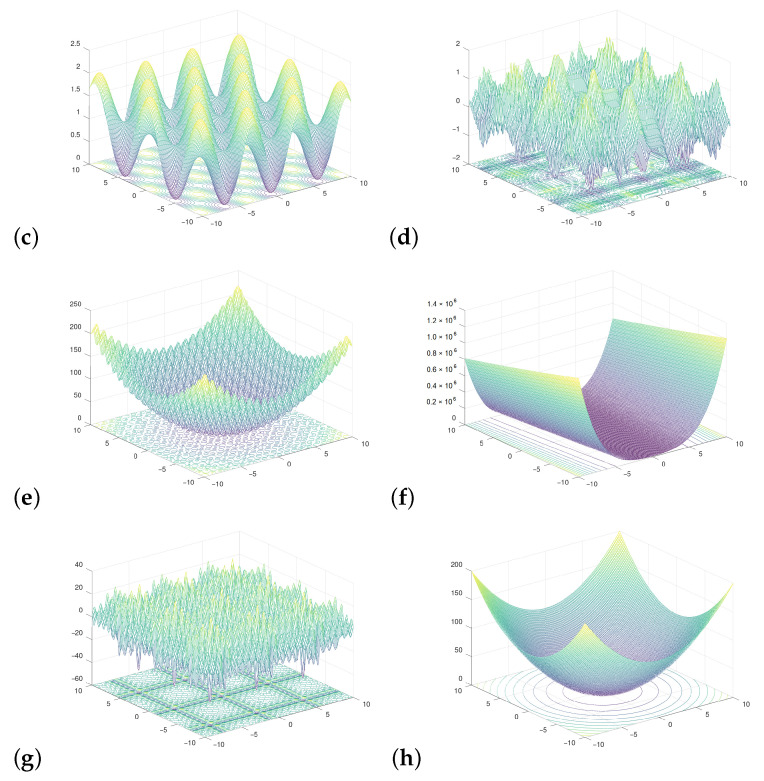
Selected fitness functions used to test the proposed algorithms: (**a**) Ackley, (**b**) Easom, (**c**) Griewank, (**d**) Michalewitz, (**e**) Rastrigin, (**f**) Rosenbrock, (**g**) Schubert, and (**h**) Sphere.

The behavior of the new version of the algorithm was validated for various FFs, shown in Figure 2. We used eight selected and representative FFs to obtain comprehensive results illustrating the performance of the proposed modifications of the conventional PSO algorithm and to compare it with the conventional approach. The results are presented for the Ackley, Easom, Griewank, Michalewitz, Rastrigin, Rosenbrock, Schubert, and the sphere FFs. Particular functions offer different features and exhibit different complexity. The simplest case is the sphere function, in which there are no local minima but only a single global minimum. Other FFs may have different numbers of local minima and as such are much more challenging for the swarm algorithms.

**Figure 3:**
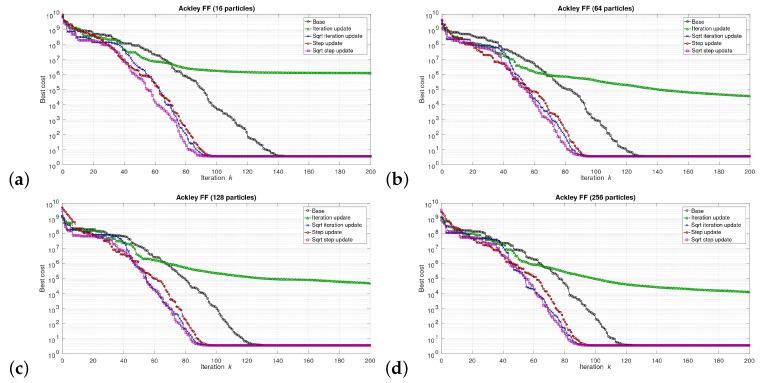
Selected results for Ackley FF for (**a**) 16, (**b**) 64, (**c**) 128, and (**d**) 256 particles.

**Figure 4:**
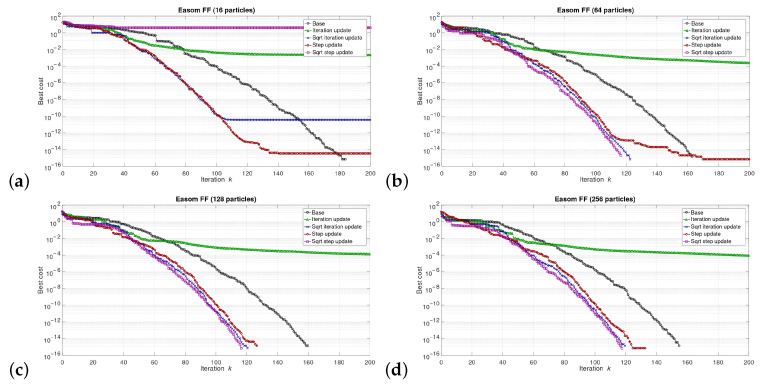
Selected results for Easom FF for (**a**) 16, (**b**) 64, (**c**) 128, and (**d**) 256 particles.

**Figure 5:**
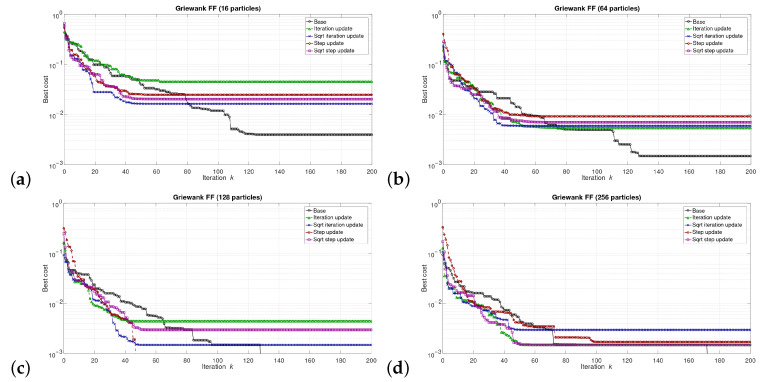
Selected results for Griewank FF for (**a**) 16, (**b**) 64, (**c**) 128, and (**d**) 256 particles.

**Figure 6:**
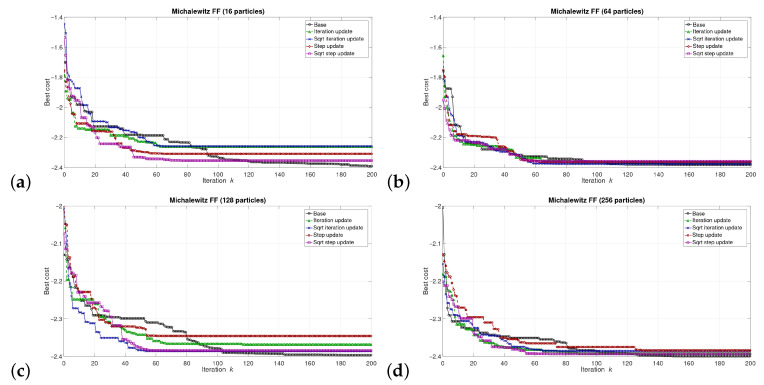
Selected results for Michalewitz FF for (**a**) 16, (**b**) 64, (**c**) 128, and (**d**) 256 particles.

**Figure 7:**
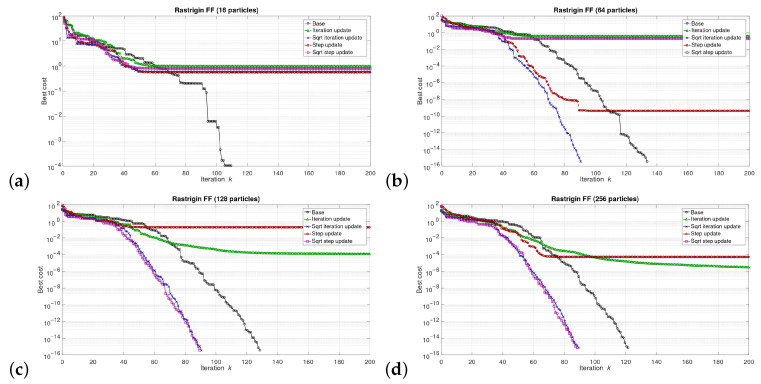
Selected results for Rastrigin FF for (**a**) 16, (**b**) 64, (**c**) 128, and (**d**) 256 particles.

**Figure 8:**
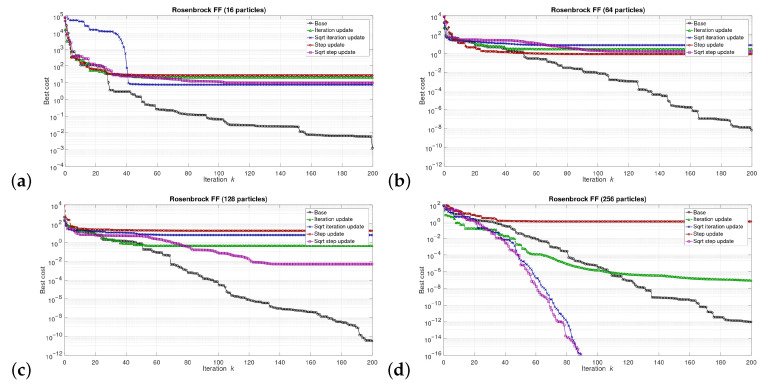
Selected results for Rosenbrock FF for (**a**) 16, (**b**) 64, (**c**) 128, and (**d**) 256 particles.

**Figure 9:**
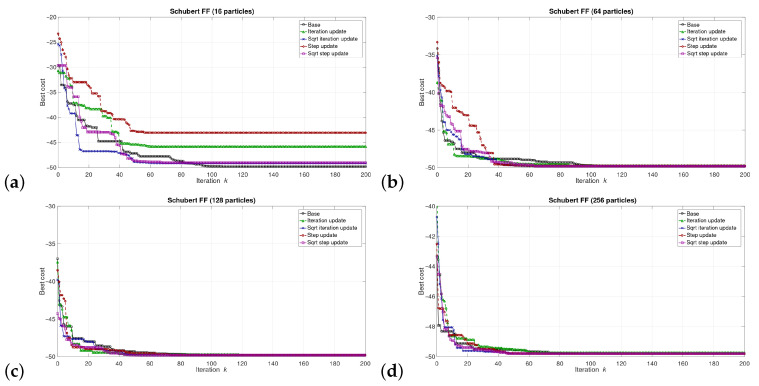
Selected results for Schubert FF for (**a**) 16, (**b**) 64, (**c**) 128, and (**d**) 256 particles.

**Figure 10:**
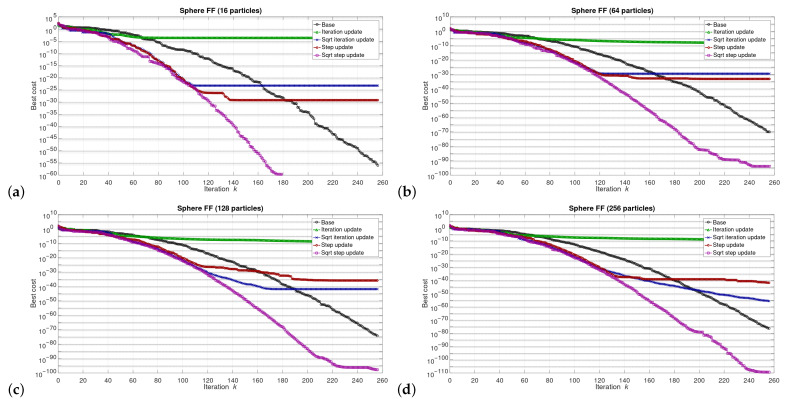
Selected results for Sphere FF for (**a**) 16, (**b**) 64, (**c**) 128, and (**d**) 256 particles.

The simulations were performed in the Matlab model for select numbers of the particles in the swarm, i.e., for 16, 64, 128, and 256 particles. We can say that the described combinations of FFs and numbers of particles are sufficiently representative to assess the proposed modifications of the algorithm. The outcomes of the simulations are shown in Figure 3, Figure 4, Figure 5, Figure 6, Figure 7, Figure 8, Figure 9 and Figure 10. Diagrams (a) in all these Figures present results for 16 particles, those in (b) present results for 64 particles, those in (c) present results for 128 particles, and those in (d) present results for 256 particles.

### 5.2. Results at the Hardware Level

**Figure 11:**
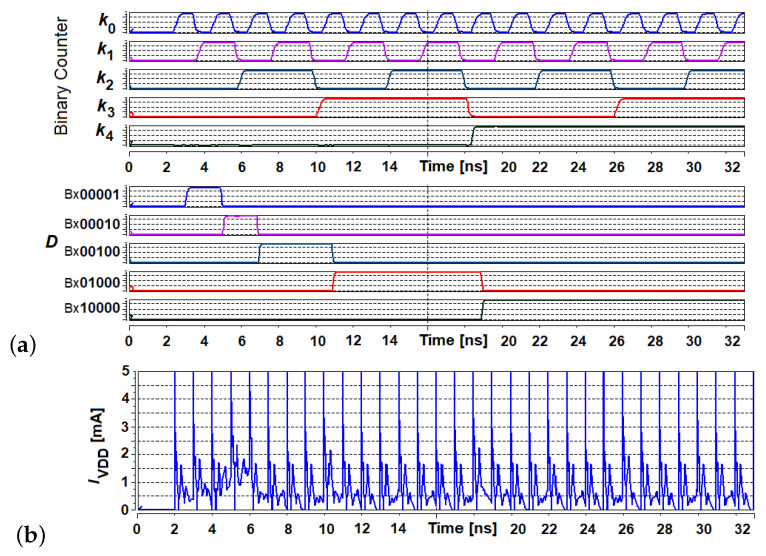

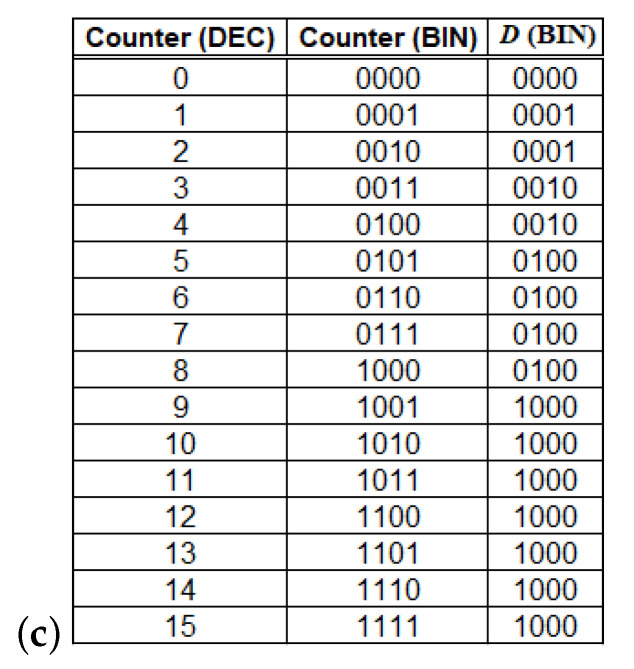
Selected simulation results illustrating the performance of the circuit shown in Figure 1: (**a**) signals representing iterations *k* and the corresponding values of the variable $D_{k}$ (in binary notation), (**b**) supply current, and (**c**) table with states of particular variables. The ‘Counter’ represents the iteration, *k*.

Figure 11 presents the selected transistor level simulation results of the circuit shown in Figure 1 as well as a table with the states of particular variables described above. This circuit throughout the *D* variable controls other blocks that are part of the proposed method.

## 6. Discussion

### 6.1. Software Level Results

The investigations carried out at the level of the software model aimed to check the performance of the proposed algorithms and thus the meaningfulness of further work at the hardware level. For this purpose, extensive simulations were conducted for different optimization problems expressed by particular FFs. In the state-of-the art literature, functions of this type are widely used to verify swarm algorithms. In our work, we present the results for eight selected FFs, with different numbers of local extremes and their different distributions in data space. For the comparison, in [48,50], only two and four FFs, respectively, were used to verify the reported DPSO algorithms.

The selected results at the model level are presented in Figure 3, Figure 4, Figure 5, Figure 6, Figure 7, Figure 8, Figure 9 and Figure 10. They are arranged in a way that particular cases focus on a specific optimization problem. Each of the figures shows the results for a single FF for different numbers of particles in the swarm. Exemplary numbers of particles that were selected are 16, 64, 128, and 256. Such an arrangement of the results allows us to observe how the size of the swarm influences its optimization abilities. For the comparison, in [48], the results are shown only for a single small number of particles.

Diagrams for particular cases show how the value of $g_{\mathrm{best}}$ variable (marked as ‘best cost’) varies as a function of the iteration *k*. Basically, two factors are important here. One of them is the achieved final value of this coefficient. The smaller the final value, the better the algorithm works in a given case. Here, however, it is worth paying attention to the need for a proper interpretation of the obtained results. In some cases, the differences in the logarithmic scale appear large, but the arbitrary final values are very small, so the difference is insignificant. For example, for the Sphere FF, the final values for each version of the algorithm vary from $10^{-110}$ to $10^{-30}$, depending on the number of particles in the swarm. The differences in terms of ratios between final values in particular cases are very large, reaching even several dozen orders of magnitude. However, as may be noticed, the ratios between the initial and the final values of the best cost factor are also very large (several dozen orders of magnitude). Therefore, it can be said that, for the Sphere FF, the final values of the best cost factor are negligible, independent of the version of the algorithm.

The second important factor is the convergence speed i.e., the number of iterations after which a given version of the algorithm reaches the optimal or acceptable solution. In all presented cases, the simulations ended after 300 iterations, although in many cases, a smaller number of iterations was sufficient to achieve satisfactory results. There may be a situation where. in all versions of the algorithm. the final values of $g_{\mathrm{best}}$ are equal, but this value was reached after a different number of iterations, *k*. For the comparison, in case of the DPSO algorithm reported in [48], the number of iterations required to complete the optimization problem is even 3000.

Each diagram in the presented particular shows the results for five versions of the algorithm. The one marked as ‘Base’ is a conventional PSO algorithm that uses a random function of $r_{1,2}$. This version is treated as a reference point against which the proposed algorithms are compared. Two of them that do not use the $\sqrt{(})$ operation in the denominator are marked as ‘Iteration update’ (equivalent to Equation (7)), and ‘Step update’ (equivalent to Equation (11)). The remaining two that use the square root operation in the denominator are labeled as ‘Sqrt iteration update’ (equivalent to Equation (6)), and ‘Sqrt step update’ (equivalent to Equation (10).

Generally, for the majority of the used FFs, the obtained results show that, with an appropriate number of particles, the proposed solutions, especially those marked as ‘Sqrt step update’, do not lead to worse optimization results. For example, for 256 particles, the proposed algorithm offered either better results or comparable with the base version for all FFs. For 64 and 128 particles, the proposed method achieved comparable or better results for all FFs except Griewank and Rosenbrock ones. For 16 particles, good results were obtained with the version marked as ’Step function’ for Easom FF. However, for 16 particles, the base version for some FFs offers a better performance.

These results show that, when the number of particles is small, then their partially random movements, which result from random values of the variables $r_{1,2}$, may lead to better results for some FFs than in the case of the proposed deterministic solutions. However, when the number of particles is higher, deterministic methods are often faster than the conventional one, offering better performance.

### 6.2. Hardware Level Results

The implementation of the PSO algorithm or its proposed modifications requires the use of different hardware components. In the case of a parallel implementation, where each particle is represented by a separate block, most of the operations are performed independently in each particle. Looking at the mathematical relationships in Section 4.3, one can observe that rather simple operations are used, such as multiplications, additions, and subtractions. Each particle is also equipped with a digital comparator realized with the use of the MBFS. It is used to compare a new (in a given iteration) value of the $p_{\mathrm{best}}$ variable with its previous value. The updated value is then stored in a memory block along with the position in which it was found. Electronic circuits representing the abovementioned operations have been developed by us earlier and verified in our former projects related to the development of AI hardware algorithms.

In addition to operations performed individually by each particle, a global operation of comparing all $p_{\mathrm{best}}$ values is also necessary in order to determine the $g_{\mathrm{best}}$ value for a given iteration *k*. Such an operation, mathematically expressed as $y=\min(x_{i})$, is often used in AI algorithms. In the PSO algorithm, it can be expressed as $g_{\mathrm{best}}\left(k\right)=\min p_{\mathrm{best},i}\left(k\right)$. In hardware, it is the most complex operation, which results from the fact that it requires the comparison of several to even several hundred multi-bit numbers stored in memory cells located in different places of the chip. If we tried to carry out such an operation as it is typically implemented by software, the problem would bring successive numbers of $p_{\mathrm{best}}$ to one comparator. It would also involve the realization of a multi-phase clock to handle this process. For this reason, in our previous work, we proposed our own solution of this type, adapted to applications in parallel energy-efficient hardware algorithms [10]. It is a simple solution working asynchronously and can handle any number of input signals in parallel in a bit-wise manner. It was implemented in a prototype chip, realized in CMOS 130 nm technology, and successfully verified by means of laboratory tests.

Taking into account our previous works in this field, in this paper, we focused on aspects that were not covered by our previous work. In this case, it is the block that substitutes the components used to generate random variables $r_{1}$ and $r_{2}$.

The proposed solution is a digital one. Therefore, we did not present detailed transistor level simulation results for the, so-called, corner analysis. It is a standard circuit verification procedure used to test the circuit before fabricating it. We performed such simulations for the temperature varying in the range from −40 to 120 C; for a supply voltage varying from 0.8 to 1.2 V; and for slow, typical, and fast transistor models. We observe that these factors translate only into the operating time of the circuit, while its behavior at the functional level (logical) remains unchanged. In Figure 11, we present the results for the slowest transistor model, which can be treated as a worst case.

The proposed algorithms are based on the use of the 1/f(k) or $1/\sqrt{f(k)}$ operation, as discussed in previous sections. However, due to a relatively small number of distinct values returned by the f(k) function, it was possible to significantly reduce the hardware complexity of the block implementing this operation. Its implementation requires only summing and bit shift operations, which are not complex in case of CMOS implementation.

The presented results show several important advantages of the proposed circuit. One of them is the high data rate. The circuits responsible for computing the *D* variable, for each iteration *k*, automatically verify the current value of this variable and updates it, if necessary. The overall process takes no more than a few nanoseconds. A similar performance is offered by the block responsible for updating the *C* value. In Figure 11 the supply current is also presented, which allows for the estimation of the energy efficiency. For a single iteration, the circuit consumes energy that does not exceed a few pJ.

The proposed circuit offers a low complexity. Depending on the assumed maximum number of the iterations, *k*, the resolution of the input and the output signals of the circuit does not exceed 9–12 bits. In the presented case, the maximum number of iterations does not exceed 300, resulting in a maximum of 9 bits of the resolution. However, since in other state-of-the art works even several thousand iterations were necessary, we also consider higher resolutions, up to 12 bits.

One of the components of the proposed circuit is the comparator (MBFS), realized as a chain of 1BFSs, in which the number equals the resolution of the input and the output signals. A single 1BFS is built of 30 transistors. Another block is the shift register, realized here as a chain of DFFs. These flip flops offer a long time data storage, suitable for the applications, in which the circuit operates with low data rates. A single DFF consists of 26 transistors. As a result, the circuit used to update the *D* variable consists of about 700 transistors. The complexity of the subcircuit responsible for updating the *C* factor (see Equation (18) is similar. It is composed of a single MBFA consisting of a chain of 1-bit full adders (1BFA) and a shift register, similar to the one used in the block updating the *D* factor.

It is worth noting that the proposed algorithm needs only a single circuit described above. As a result, the hardware complexity of this block is negligible when compared with the remaining components used in the algorithm. It eliminates the need to equip each particle in the swarm with a dedicated block for generating random values of the *r* factors.

The advantage of this approach is also visible when comparing it with other DPOS algorithms reported in the literature. For example, in the DPSO algorithm and its variants presented in [46,47,48,49,50], the blocks generating the random values are replaced with deterministic blocks determining the trajectories of particular particles in the swarm. They require computation of eigenvalues of the matrices expressing the updates of the movement parameters of particular particles. It requires additional mathematical operations, including the square root. Additionally, it involves the calculation of trigonometric functions. Therefore, this solution is much more challenging for the realization at the transistor level. The authors of that work, moreover, did not propose such an implementation but only provided such a possibility as a motivation for their investigations.

In [51], the random factors were eliminated, which means that the values of $c_{1,2}$ and $r_{1,2}$ were simply set to 1. It is difficult to assess the performance of this algorithm, as the authors of this work did not present comparable model-level investigations.

## 7. Conclusions

The aim of this work was to present a novel approach to the implementation of the PSO algorithm. The proposed approach facilitates transistor implementation of the algorithm. At the same, time the system level performance of the algorithm for an appropriate number of particles in the swarm is even better than in case using the conventional algorithm. We proposed a parallel implementation, in which each particle in the swarm is realized as a separate block. In this case, any optimization of the computation scheme leads to substantial savings in hardware resources.

During the implementation of the deterministic swarm algorithm, new hardware solutions were proposed that may have a wider application than just in this one algorithm. One of them is a system that allows for the approximation of the $1/\sqrt{\left(\right)}$ operation. In a similar way, one can build a system approximating the $\sqrt{\left(\right)}$ operation. All that is needed is different coefficients in the *C* variable set.

The presented work is one of the steps of a larger project realized at our university. We already designed a prototype chip in CMOS 130 nm technology, containing other components of the PSO algorithm, such as the circuit responsible for computing the global best value in the swarm. The modifications introduced in this work can be quickly introduced to a final solution that is now under development.

## Data Availability

The data presented in this study are available upon request from the corresponding author.

## Abbreviations

The following abbreviations are used in this manuscript:

1BFA	1-bit full adders
1BFS	1-bit full subtractor
ABC	Artificial Bee Colony
ACO	Ant Colony Optimization
BA	Bat Algorithm
BFO	Bacterial Foraging Optimization
DFF	D-flip flops
FF	Fitness Function
LUT	Look-Up-Table
MBFA	multi-bit Full Adder
MBFS	Multi-bit Full Subtractor
MSB	Most Significant Bit
PSO	Particle Swarm Optimization
SVM	Support Vector Machine
